# Ibrutinib‐induced severe liver injury

**DOI:** 10.1002/ccr3.881

**Published:** 2017-03-15

**Authors:** Amara G. Nandikolla, Olga Derman, Deborah Nautsch, Qiang Liu, Hatef Massoumi, Sangeetha Venugopal, Ira Braunschweig, Murali Janakiram

**Affiliations:** ^1^ Department of OncologyMontefiore Medical Center/Albert Einstein College of MedicineBronxNew YorkUSA; ^2^ Department of PathologyMontefiore Medical Center/Albert Einstein College of MedicineBronxNew YorkUSA; ^3^ Department of HepatologyMontefiore Medical Center/Albert Einstein College of MedicineBronxNew YorkUSA; ^4^ Department of MedicineMontefiore Medical CenterBronxNew YorkUSA

**Keywords:** Chronic lymphocytic leukemia, ibrutinib, liver injury, Richter's syndrome, tyrosine kinase inhibitors

## Abstract

Ibrutinib, an inhibitor of the Bruton's tyrosine kinase of the B‐cell receptor pathway, is an effective therapeutic agent for B‐cell lymphomas. As these drugs are novel, long‐term or rare adverse events are not yet known. We report the first case of ibrutinib‐induced severe liver injury in a patient with relapsed/refractory CLL.

## Introduction

Ibrutinib is an oral inhibitor of Bruton's tyrosine kinase. It is approved for a variety of B‐cell‐related hematological malignancies including relapsed or refractory chronic lymphocytic leukemia (CLL). Grade 3 adverse events reported in the clinical trials included atrial fibrillation, bleeding, thrombocytopenia, and neutropenia; however, severe hepatic impairment was not reported. In this first‐in‐literature case, we report on severe cholestatic hepatic injury caused by ibrutinib in a patient with relapsed and refractory CLL.

## Case Presentation

A 62‐year‐old African American male was diagnosed with Rai stage I CLL (ZAP 70+, CD38+, deletion 11q+) in 2005 on a cervical lymph node biopsy. He remained on observation until 2009. At that time, his cervical lymphadenopathy worsened, and he presented with new constitutional symptoms of weight loss, fatigue, and night sweats. Therefore, he was treated with six cycles of FCR (fludarabine, cyclophosphamide, and rituximab). He achieved a complete response (CR) and remained under surveillance until 2014 when he presented with rapidly expanding right‐sided cervical lymphadenopathy compressing on his esophagus and new lymphocytosis of 20,000/*μ*L. Lymph node biopsy was consistent with Richter's transformation to diffuse large B‐cell lymphoma (DLBCL). Targeted genomic analysis showed missense and frameshift mutations in the PRDM1 and SPEN genes.

After the diagnosis of Richter's transformation, he was treated with six cycles of R‐CHOP chemotherapy (rituximab, cyclophosiphamide, doxorubicin, vincristine, prednisone) followed b a autologous stem cell transplant with BEAM conditioning (carmustine, etoposide, cytarabine, melphalan) which was completed in March 2015. Post transplant, he remained on close clinical follow‐up. In August 2015, he presented with fatigue and abdominal pain and was found to have bulky intra‐abdominal lymphadenopathy. He was treated with four cycles of R‐ICE (rituximab, ifosfamide, carboplatin, and etoposide), but the disease remained refractory. As ibrutinib has shown activity in Richter's transformation, ibrutinib 420 mg was started 4 weeks after the last cycle of R‐ICE. Throughout his entire course including the different chemotherapy regimens since diagnosis and in the 6 days before initiation of ibrutinib, the patient's liver function tests (LFTs) remained normal.

**Figure 1:**
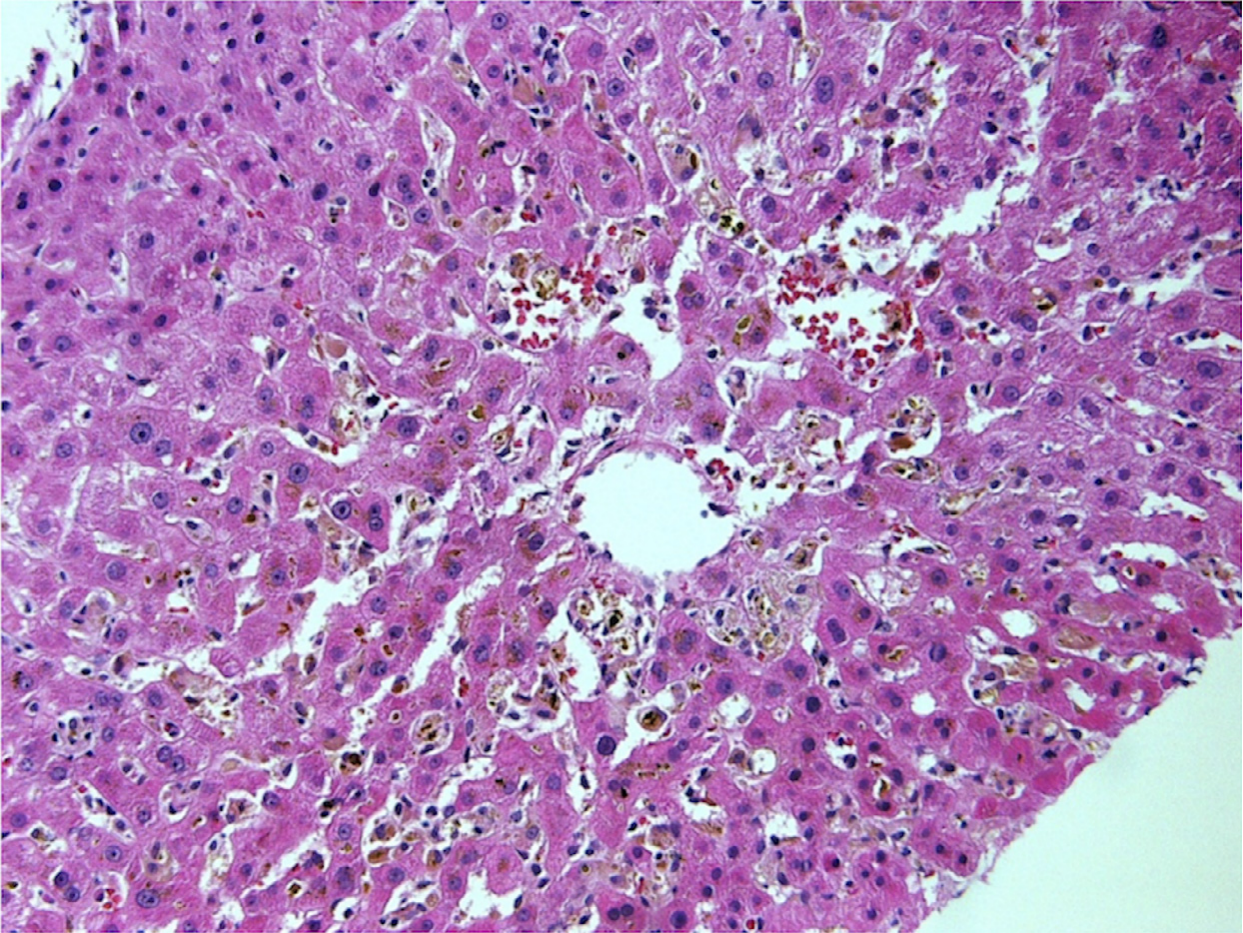
Liver biopsy: There is marked hepatocellular and canalicular cholestasis around the central veins associated with hepatocyte injury and acidophil bodies. These findings are compatible with a cholestatic pattern of drug‐induced liver injury.

Two weeks after initiation of ibrutinib, routine blood work revealed new onset LFT abnormalities (Table 1) and his only reported symptom was dark‐colored urine for 3 days. At the time of ibrutinib initiation, the patient was not on any medications including herbal, dietary supplements, or over‐the‐counter medications. A complete infectious workup including serologic testing for cytomegalovirus, Epstein‐Barr virus, hepatitis viruses (hepatitis C virus antibody, hepatitis B surface antigen, hepatitis B core antibody, and hepatitis E IgG and IgM), blood cultures, screening for autoimmune diseases including ANA (antinuclear antigen), antimitochondrial antibodies, antismooth muscle antibodies, and antiliver kidney microsomal antibodies (anti‐LKM antibody) were all negative. Imaging studies including an ultrasound of the liver and CT scan of the abdomen did not show any changes in his disease status compared with prior imaging, nor any evidence of obstruction at the level of the biliary tree was seen. As drug‐induced liver injury was suspected, ibrutinib was stopped and to delineate further he underwent two liver biopsies, as his LFTs never normalized. The liver biopsy showed centrilobular intrahepatic and canalicular cholestasis, ceroid‐laden macrophages (highlighted on PAS‐D stain), and rare apoptotic bodies, which was consistent with a drug‐induced liver injury (Fig. 1). His LFTs down trended 3 months after stopping ibrutinib but did not normalize (Table 1) and he continued without further therapy. Unfortunately, he passed away due to the progression of CLL and a decline in his performance status in May 2016.

**Table 1:** Trend of liver function test after stoppin ibrutinib

	Bilirubin total	Bilirubin direct	SGOT	SGPT	Alkaline phosphatase
Normal values	0.2–1.1 MG/DL	0.1–0.3 mg/dL	11–42 U/L	0–20 U/L	49–158 U/L
12/8/2015	0.5	0.1	18	22	66
12/29/2015	7.1	5.5	448	743	486
1/5/2015	9.5	6.8	448	1245	344
1/18/2016	23.8	15.9	23	39	317
2/8/2016	29.4	20.0	77	96	652
2/16/2016	35.2	23.4	226	191	664
3/4/2016	25.5	17.3	75	71	618
3/8/2016	18.0	11.3	88	97	632

## Discussion

Ibrutinib is a small molecule inhibitor of the enzyme Bruton's tyrosine kinase (BTK). The B‐cell receptor pathway is important in the development and maturation of B‐ cells. BTK is a downstream receptor critical for B‐cell receptor signaling 1. Ibrutinib is an effective therapy in multiple lymphoid malignancies including relapsed or refractory CLL, CLL with 17p deletion, mantle cell lymphoma, Waldenstrom's macroglobulinemia, and Richter's transformation 2, 3, 4, 5, 6, 7.

In clinical studies with ibrutinib, no reports of liver injury following drug administration were noted 3, 8, 9. The most common toxicities reported are grades 1–2 including diarrhea, nausea, vomiting, decreased appetite, dyspepsia, fatigue, myalgia, and arthralgia. Grade 3–4 toxicities included neutropenia, thrombocytopenia, bleeding, and atrial fibrillation 9, 10. More recently, a small case series of pneumonitis as an adverse effect has been reported 11.

Ibrutinib is metabolized in the liver by cytochrome P450, CYP3A, and to a minor extent by CYP2D6. Genomic analyses of our patient's peripheral blood showed PRDM1 and SPEN mutations, both of which are unlikely to be somatic mutations involved in the liver metabolism of the drug. As per the prescribing information, a hepatic impairment study was performed, and they found that the area under the curve after administration of single dose of ibrutinib was increased by 2.7‐, 8.2‐, and 9.8‐fold in subjects with mild (Child‐Pugh class A), moderate (Child‐Pugh class B), and severe (Child‐Pugh class C) hepatic impairment, respectively, compared to subjects with normal liver function. Therefore, for patients with Child‐Pugh score A, the recommended dose is 140 mg daily and should be avoided in patients with Child‐Pugh classes B and C. Grade 4 increase in LFTs has been reported in a healthy subject after a dose of 1680 mg. Based on this, we could conclude that ibrutinib accumulates in an impaired liver and could also cause liver injury.

Drug‐induced liver injury is mostly idiosyncratic and can present clinically as hepatocellular, cholestatic, or with mixed etiology 12. Mechanisms of hepatotoxicity can be variable. They include immunologic mechanisms, oxidative stress from metabolites, genetic variations in drug metabolism, and drug‐induced mitochondrial dysfunction 13. Patients usually fully recover upon drug discontinuation, but the time period can vary between 30 days to a year, especially for patients with severe cholestasis 14.

Hepatotoxicity to a variable degree is seen in various tyrosine kinase inhibitors (TKIs). The severity may differ, although fatality from liver failure is relatively rare. As the toxicity varies among the same class, the FDA requires monitoring of LFTs for the TKIs such as crizotinib, imatinib, lapatinib, pazopanib, ponatinib, regorafenib, and sunitinib 13. Genetic variations in metabolism have been described with lapatinib and pazopanib. In pazopanib, hyperbilirubinemia is associated predominantly with the UGT1A1*28 polymorphism and may also have an association with HFE polymorphisms. Liver injury caused by lapatinib has been associated with carriers of the allele DRB1*07:01/DQA1*02:01 in the class II HLA locus 15.

Our patient had normal LFTs before initiation of ibrutinib, negative workup for infectious and autoimmune etiologies of liver injury, and two liver biopsies consistent with drug‐induced liver injury, leading to the conclusion that ibrutinib was the cause of his liver injury. To our knowledge, this case is the first reported case of ibrutinib‐induced severe liver injury. The exact pathophysiology of this liver toxicity is not yet known and requires closer monitoring and investigation in both the phase 4 setting and current clinical trials with ibrutinib.

## Authorship

AGN: participated in acquisition of relevant literature, manuscript writing and editing, revising critical and intellectual content, table creation, table editing, and final approval of the version to be published. OD, QL, DN, HM, and IB: participated in acquisition of relevant literature and editing and revising critical and intellectual content. MJ: participated in conception and design of the work, acquisition of relevant literature, manuscript editing, revising critical and intellectual content, and final approval of the version to be published.

## Conflict of Interest

None declared.
